# Circulating Cell-Free DNA in Metabolic Diseases

**DOI:** 10.1210/jendso/bvaf006

**Published:** 2025-01-15

**Authors:** Alessio Pollastri, Peter Kovacs, Maria Keller

**Affiliations:** Medical Department III—Endocrinology, Nephrology, Rheumatology, University of Leipzig Medical Center, Leipzig 04103, Germany; Helmholtz Institute for Metabolic, Obesity and Vascular Research (HI-MAG) of the Helmholtz Center Munich at the University of Leipzig and University Hospital Leipzig, Leipzig 04103, Germany; Medical Department III—Endocrinology, Nephrology, Rheumatology, University of Leipzig Medical Center, Leipzig 04103, Germany; Deutsches Zentrum für Diabetesforschung e.V., Neuherberg 85764, Germany; Medical Department III—Endocrinology, Nephrology, Rheumatology, University of Leipzig Medical Center, Leipzig 04103, Germany; Helmholtz Institute for Metabolic, Obesity and Vascular Research (HI-MAG) of the Helmholtz Center Munich at the University of Leipzig and University Hospital Leipzig, Leipzig 04103, Germany

**Keywords:** cell-free DNA, epigenetics, obesity, diabetes, MAFLD

## Abstract

Metabolic diseases affect a consistent part of the human population, leading to rising mortality rates. This raises the need for diagnostic tools to monitor the progress of these diseases. Lately, circulating cell-free DNA (cfDNA) has emerged as a promising biomarker for various metabolic diseases, including obesity, type 2 diabetes, and metabolic-associated fatty liver disease. CfDNA is released from apoptotic and necrotic cells into the bloodstream and other body fluids, and it retains various molecular signatures of its tissue of origin. Thus, cfDNA load and composition can reflect tissue pathologies and systemic metabolic dysfunctions. In addition to its potential as a diagnostic biomarker, interest in cfDNA derives from its recently discovered role in adipose tissue inflammation in obesity. This review discusses detection methods and clinical significance of cfDNA in metabolic diseases.

Metabolic diseases, including obesity, type 2 diabetes (T2D), and metabolic-associated fatty liver disease (MAFLD), are global health concerns with rising prevalence and mortality rates. These conditions are characterized by chronic low-grade inflammation, insulin resistance, and dysregulated lipid and glucose metabolism [1]. Circulating cell-free DNA (cfDNA) consists of small DNA fragments—typically 160 to 180 base pairs (bp) in length—released into the bloodstream from dying or stressed cells, providing insights into the genetic and epigenetic changes occurring in the tissue of origin. In the second half of the last century, it was shown that several diseases, such as systemic lupus erythematosus and cancer, led to elevated cfDNA levels in human blood [2, 3]. Since then, many studies have investigated and demonstrated that alterations in blood cfDNA concentration were associated with specific pathological conditions. In fact, while cfDNA fragments in healthy individuals were shown to be predominantly of hematopoietic origin, several pathological conditions can cause augmented apoptotic events, resulting in higher amounts of cfDNA being released from the affected tissues [4, 5].

In cancer patients, cfDNA harbors specific mutations that are found in the patient's tumor cells, and it is thus referred to as circulating tumor DNA (ctDNA) [6]. This peculiar characteristic of cancer-associated cfDNA was first recognized in 1994, when mutated *KRAS* sequences detected in plasma or serum from patients affected by pancreatic carcinoma were also found in the tumors of these patients [7]. Since then, increasing evidence of ctDNA potential as biomarkers has been collected, and it has not been limited to the bloodstream, but has involved other body fluids as well, such as urine and saliva [8, 9]. The possibility of exploiting different easily accessible fluids to detect the presence of specific disease signatures makes cfDNA's prognostic and diagnostic potential even broader. In fact, specific tumors might be more easily identifiable in body fluids other than plasma, like saliva in the case of head and neck squamous cell carcinomas or urine in the case of bladder cancer [10, 11]. Advances and challenges of cancer-related cfDNA studies have been exhaustively reported elsewhere [12-16] and will not be further discussed in this review.

CfDNA applications are not limited to cancer studies. Since Lo et al [17] found fetal cfDNA in maternal plasma and serum, several groups have explored how to use cfDNA in noninvasive prenatal screening, for instance to detect aneuploidy [18] or more recently phenylketonuria [19]. CfDNA applications have already revealed great potential in cancer and prenatal screening, to the point that cfDNA testing based on specific gene panels is now the object of various clinical trials, and it has already been proven to be valuable within some national health systems [20, 21]. However, these are not the only areas where cfDNA have been investigated, and metabolic disorders represent another emerging field in this regard. Thus, this review will explore the current understanding of cfDNA in metabolic diseases, highlighting its potential as a biomarker in clinical diagnostics (Fig. 1).

**Figure 1.**
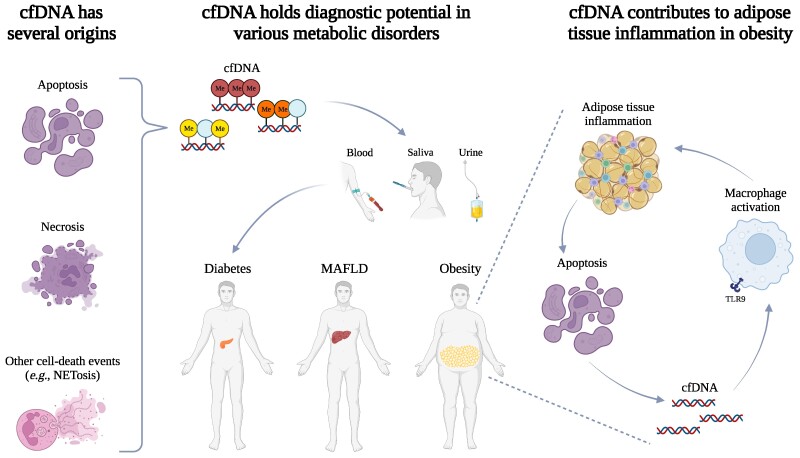
Circulating cell-free DNA (cfDNA) consists of small DNA fragments that originate from apoptosis, necrosis, or other cell-death events like NETosis, and that are released into the blood and other body fluids. As cfDNA retains epigenetic signatures of its tissue of origin, it can be isolated from blood, urine, or saliva and can potentially be used to gain insights into the epigenetic mechanisms underlying specific metabolic disorders, such as diabetes, obesity, and metabolic-associated fatty liver disease (MAFLD). In addition to having potential as a diagnostic tool, cfDNA was shown to contribute to adipose tissue inflammation in obesity, meaning cfDNA is not only a biomarker, but it actively contributes to the pathogenesis of this disorder. This figure was created with BioRender.com.

To provide up-to-date information on the discussed topic, the PubMed database was used to find scientific articles. The search was performed with the keyword “cfDNA” plus the AND conjunction and one of the following key words: “obesity,” “diabetes,” “T1D,” “T2D,” “MAFLD,” “NAFLD,” “metabolic disorders.” Also, to filter for information specifically related to metabolic disorders, further searches were performed with the key words “cancer” and “prenatal screening” added via the NOT conjunction.

## Origins and Characteristics of Circulating Cell-Free DNA

CfDNA primarily originates from cell death, mainly through apoptosis or necrosis, but other processes like NETosis (NET—neutrophil extracellular traps) can also contribute to their release [22]. Typically, cfDNA fragment length was found to be in the 160 to 180 bp range, with a dominant peak at 166 to 167 bp, thus corresponding to the length of a chromatosome (ie, nucleosome plus linker histone) [23-25]. This size distribution most likely reflects the protection from nuclease activity provided to cfDNA by its association with proteins, and it supports the origin of cfDNA as mainly deriving from enzymatic cleavage of genomic DNA in apoptotic cells [23, 26], although longer fragments can also be present, likely originating from necrotic events [27]. Since cfDNA retains the molecular characteristics of its tissue of origin, such as fragmentation and methylation patterns, it can provide valuable insights into epigenetic mechanisms underlying various pathological states [28, 29].

## Cell-Free DNA in Obesity

There is emerging evidence for the role of cfDNA in metabolic diseases. Haghiac et al [30] reported higher cfDNA levels in obese pregnant women compared to lean pregnant women. Since then, several groups have investigated the relation between cfDNA blood load and obesity-related anthropometric traits (eg, body mass index, body fat percentage, or waist-to-hip ratio), but whether a direct relation exists remains controversial [31-33]. Nevertheless, several studies have demonstrated that cfDNA levels positively correlated with metabolic parameters often associated with obesity, such as the homeostatic model assessment of insulin resistance and C-reactive protein [32, 34]. In accordance with this finding, the release of cfDNA has been associated with inflammatory diseases, and at the same time, obesity is known to be characterized by excessive adipose tissue expansion and chronic low-grade inflammation, which contribute to insulin resistance and metabolic dysregulation [35]. When looking at epigenetic signatures, several cfDNA differentially methylated regions were identified comparing lean and obese Göttingen minipigs [33], once again highlighting the diagnostic potential of this biomarker.

In the context of a mechanistic role in obesity, it was demonstrated that cfDNA derived from adipocytes can act through the toll-like receptor 9 signaling pathway to activate macrophages [35]. Further in vitro studies revealed that cfDNA decreased adiponectin secretion in 3T3-L1 adipocytes, likely acting through various pattern recognition receptors [36]. These findings suggest that cfDNA contributes to the inflammatory state that characterizes obesity, although the magnitude of cfDNA action in this context has not yet been elucidated.

Overall, these studies highlight cfDNA’s potential as a noninvasive biomarker for assessing adipose tissue cell death rate and inflammation in obesity, and suggest that these DNA fragments can to some extent contribute to the pathophysiology of this condition.

## Cell-Free DNA in Diabetes

T2D is characterized by insulin resistance and β-cell dysfunction, and lately, chronic systemic inflammation has been shown to be involved in disease progression [37]. The use of cfDNA-based tools to identify T2D complications has been the object of several studies [38-41]. These works exploit the 5hmC-Seal technology [42], which enables biotin labeling of 5-hydroxymethylcytosine (5hmC)-containing cfDNA fragments and their subsequent polymerase chain reaction (PCR) amplification and next-generation sequencing analysis. Their results suggest that the levels of specific 5hmC markers might allow us to differentiate between T2D patients with and without vascular complications, such as diabetic retinopathy and diabetic kidney disease.

CfDNA emerged as a biomarker for type 1 diabetes (T1D) as well. Akirav et al [43] developed an assay based on methylation-sensitive primers to distinguish β-cell– and non-β-cell–derived cfDNA, as they found CpG sites in the *INS* gene specifically unmethylated in β cells. Later on, the efficiency of the assay was improved by including the methylation status of CpGs in other genes like chromatin target of PRMT1 (*CHTOP*) [44]. Although changes in unmethylated *INS* cfDNA levels did not allow the differentiation between patients with or without T2D [45], they were associated with T1D onset [43, 46]. As promising as these initial findings are, there is the need to validate and optimize the (un)methylation detection assays so as to obtain more reliable and reproducible results across different laboratories. Speake et al [47] started addressing this need by comparing the performances of 3 unmethylated insulin DNA assay, proving them all effective in quantifying circulating unmethylated *INS* levels.

## Cell-Free DNA in Metabolic-Associated Fatty Liver Disease

MAFLD encompasses a spectrum of liver conditions ranging from simple steatosis to metabolic dysfunction–associated steatohepatitis and cirrhosis. As progression of MAFLD is strictly dependent on inflammatory mechanisms, cfDNA has been proposed as a noninvasive biomarker for liver damage in MAFLD, reflecting hepatocyte apoptosis and necrosis [48]. Although cfDNA concentration has been found to correlate with established MAFLD markers, the relation between cfDNA levels and MAFLD progression is yet to be fully demonstrated and remains elusive [48, 49]. Interestingly, cfDNA methylation levels in cfDNA could be used, together with other biomarkers, to detect the presence of metabolic dysfunction–associated steatohepatitis, while plasma levels of the macroH2A1.2 histone variant appear to be higher in patients with MAFLD [49]. These results suggest that cfDNA could provide a noninvasive method for assessing liver fibrosis and monitoring disease progression in MAFLD patients.

## Cell-Free DNA Analysis Workflow

The emerging potential applications of cfDNA as a biomarker have inspired numerous research groups to promote and intensify their efforts in this field. The growing interest in the detection and quantification of cfDNA as potential diagnostic tool raises the urgency to standardize both preanalytical and analytical steps of cfDNA processing. In regard to the first aspect, Meddeb et al [50] addressed the challenges that come between patient identification and actual cfDNA analysis, including blood collection, plasma preparation, and cfDNA extraction. Other research groups have dealt with some of these issues as well, providing the foundation to create common and validated standard procedures to be followed in different laboratories, so as to obtain more reliable and reproducible data [51, 52]. One of the main concerns is plasma isolation, as it is fundamental that cell damage and consequent genomic DNA release be avoided. It has been established that the optimal procedure consists of 2 centrifugation steps to be performed within 4 hours from blood sampling. In particular, blood is first centrifuged at 1 200*g* for 10 minutes to prevent blood cells destruction, and the resulting plasma is then further centrifuged at 16 000*g* for 10 minutes to remove any cell debris [53].

**Figure 2.**
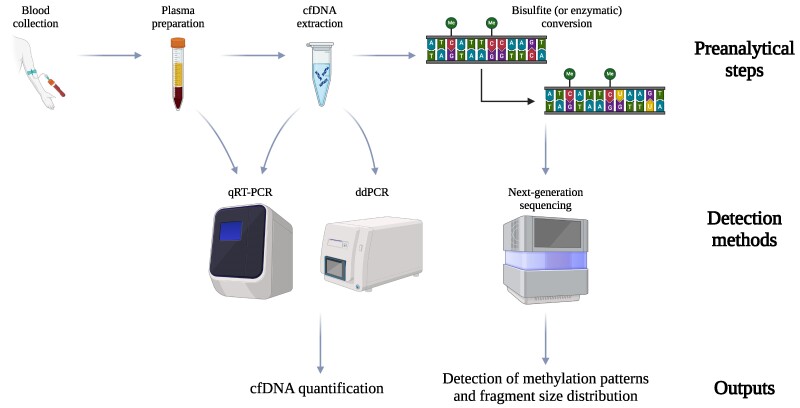
The first steps of circulating cell-free DNA (cfDNA) analysis workflow include blood collection and subsequent centrifugation to separate the cfDNA-containing plasma from the corpuscular part of blood. Directly following cfDNA extraction, quantitative real-time polymerase chain reaction (qRT-PCR) and digital droplet PCR (ddPCR) can be used to quantify the amount of circulating cfDNA, although qRT-PCR methods that do not even require prior extraction are being developed. In addition, bisulfite (or more recently enzymatic) conversion and subsequent sequencing provides information concerning cfDNA methylation patterns and fragment size distribution. This figure was created with BioRender.com.

Various quantification methods have been engaged in cfDNA studies (Fig. 2). The fastest approaches aimed at cfDNA quantification are PCR-based methods, which can be performed right after the extraction, with no intermediate processing needed. These methods include quantitative real-time PCR (qRT-PCR), which is a reliable yet simple and cost-effective technique, and droplet digital PCR, which offers higher sensitivity and precision [34, 54]. Recently, PCR-based methods that do not even require prior cfDNA extraction from blood have been developed, so as to avoid DNA loss during this step [55]. Alternatively, electrophoresis-based methods (eg, Bioanalyzer) represent a reliable approach to obtain data about cfDNA size and concentration. These methods offer the advantage of enabling quality and integrity control of the extracted DNA fragments, thus allowing for genomic DNA contamination checking [56, 57]. In case more detailed information (such as methylation patterns and fragment size distribution) is required, sequencing-based approaches are the techniques of choice. These methods require library preparation steps like bisulfite conversion (necessary for methylation detection), which usually causes substantial cfDNA chemical degradation. Ørntoft et al [58] highlighted this issue by comparing the conversion efficiency of 12 commercially available kits, showing clear performance differences between the various kits. Enzymatic conversion is emerging as an alternative to bisulfite conversion [59, 60], as it enables a lower degradation rate of the starting cfDNA material, but this methodology has not been implemented in the field of metabolic disorder yet. Nevertheless, next-generation sequencing allows the determination of cfDNA methylation profiling, making it possible for researchers to determine the cfDNA tissues of origin. To retrieve this information, various tissue deconvolution methods have been developed, such as the ones by Moss et al and Caggiano et al, which are designed to decompose methylation array data and whole-genome bisulfite sequencing reads, respectively [61, 62]. These methods use a reference methylation panel to estimate the relative abundance of the various cell and/or tissue types cfDNA originates from, therefore enabling the obtaining of information about cfDNA composition under specific pathological conditions.

## Concluding Remarks

In this brief review, we have presented the current state of cfDNA research in metabolic disorders, focusing on obesity, T2D, and MAFLD. CfDNA represents a promising diagnostic tool, not only in the cancer and prenatal testing fields, where cfDNA detection methods are efficiently being established, but in metabolic disorders as well. The difference in cfDNA research advancement between cancer- and metabolic disorder–related studies is likely due to the fact that cancer diagnostics can rely on the detection of specific sequence mutations to discriminate between cfDNA derived from cancer cells and that derived from healthy cells. On the other hand, in the field of metabolic disorders, it is mainly the epigenetic signature of cfDNA that holds valuable information, and the characterization of these markers is more challenging. In fact, most atlases used for tissue deconvolution do not yet contain clearly distinctive epigenetic signatures for all the different cell types, making it complicated to apply these technologies in some specific fields. Even so, the possibility to access DNA fragments originating from pathology-affected tissues directly from the blood makes this biomarker an incredibly interesting tool. Furthermore, as DNA extraction methods from other body fluids like urine (in which DNA degradation is quicker) are being implemented [63], it would be interesting to verify whether cfDNA measurements from these sources could be useful in the field of metabolic diseases as well. As mentioned earlier, standardized preanalytical and analytical procedures are needed to produce reliable and comparable results, which will allow us to confidently identify specific cfDNA signatures reflecting the pathological state of patients. In addition, the implementation of sequencing techniques and subsequent deconvolution methods will facilitate the determination of cfDNA methylation markers associated with disease progression and complications.

Finally, recent works on how cfDNA also directly contributes to inflammation, especially in the context of obesity, have been reported previously. These results are still very preliminary, and the extent of cfDNA’s role in inflammation is far from clear. Nevertheless, further investigations on the biological activity of circulating DNA fragments might lead to the discovery on new potential targets of anti-inflammatory drugs.

## Data Availability

Data sharing is not applicable to this article as no data sets were generated or analyzed during the present study.

## Abbreviations

5hmC: 5-hydroxymethylcytosine
bp: base pairs
cfDNA: cell-free DNA
ctDNA: circulating tumor DNA
MAFLD: metabolic-associated fatty liver disease
NAFLD: non-alcoholic fatty liver disease
NET: neutrophil extracellular traps
qRT-PCR: quantitative real-time polymerase chain reaction
T1D: type 1 diabetes
T2D: type 2 diabetes
